# Cryoballoon vs. open irrigated radiofrequency ablation for paroxysmal atrial fibrillation: long-term FreezeAF outcomes

**DOI:** 10.1186/s12872-017-0566-6

**Published:** 2017-05-25

**Authors:** Armin Luik, Kevin Kunzmann, Patrick Hörmann, Kerstin Schmidt, Andrea Radzewitz, Peter Bramlage, Thomas Schenk, Gerhard Schymik, Matthias Merkel, Meinhard Kieser, Claus Schmitt

**Affiliations:** 1 Medizinische Klinik IV, Städtisches Klinikum Karlsruhe, Academic Teaching Hospital of the University of Freiburg, Karlsruhe, Germany; 2 Institute of Medical Biometry and Informatics, University of Heidelberg, Heidelberg, Germany; 3 Institute for Pharmacology and Preventive Medicine, Mahlow, Germany

**Keywords:** Atrial fibrillation, Catheter ablation, Radiofrequency, Cryoablation, Cryoballoon, Pulmonary vein isolation

## Abstract

**Background:**

Effective treatment of paroxysmal atrial fibrillation (AF) is essential for reducing the risk of stroke and heart failure. Cryoballoon (CB) ablation has been developed as an alternative to the use of radiofrequency (RF) energy for electrical isolation of the pulmonary veins. Herein, we provide long-term data regarding the efficacy of CB ablation in comparison to RF.

**Methods:**

FreezeAF was a randomised non-inferiority study comparing CB ablation with RF ablation for the treatment of patients with drug-refractory paroxysmal AF. Procedural success for the long-term follow-up (30 months) was defined as freedom from AF with an absence of persistent complications.

**Results:**

Of the 315 patients that were randomised and received catheter ablation, 292 (92.7%) completed the 30-month follow-up (147 in the RF group and 145 in the CB group). The baseline characteristics of the RF and CB groups were similar. Single-procedure success was achieved by 40% of patients in the RF group and 42% of the CB group (*p* < 0.001 for non-inferiority). When including re-do procedures in the analysis, the multiple procedure success rate was 72% in the RF group and 76% in the CB group.

**Conclusion:**

The data provide long-term evidence that CB ablation is non-inferior to RF ablation, with high proportions of patients reporting freedom from AF 30 months after the index procedure.

**Trial registration:**

ClinicalTrials.gov Identifier: NCT00774566; first registered October 16, 2008; first patient included October 20, 2008.

## Background

Atrial fibrillation (AF) is associated with an increased risk of stroke, heart failure, and death [1, 2]. While antiarrhythmic drugs (AADs) benefit some patients, they are often inadequate and have been associated with intolerance due to adverse events [3]. The development of catheter ablation has provided patients with an alternative solution, one which has shown efficacy as both a first- and second-line treatment for AF [4]. Indeed, guidelines now state that drug-refractory paroxysmal AF is a class I, level A indication for catheter ablation [5]. The most common approach to this is the electrical isolation of the pulmonary veins (PV) by the creation of circumferential lesions around the right and left PV ostia. Such lesions have most commonly been formed using point-by-point application of radiofrequency (RF) energy. However, this procedure is complex and has been associated with serious complications, including cardiac tamponade [6]. A more recently developed alternative is the application of cryo-energy via a balloon catheter. This technique enables the lesion to be produced in a single step, thereby potentially reducing the length of the procedure. However, it also requires more extensive use of fluoroscopy and can cause phrenic nerve injury as well as cardiac tamponade [7, 8].

Good outcomes after cryoballoon (CB) ablation have been demonstrated, with high proportions of patients remaining free from AF after long-term follow-up [7, 9, 10]. A number of studies have compared RF with CB ablation for the treatment of paroxysmal AF, and have found similar procedural success rates [6, 11–15]. However, there are few studies that are both randomised and involve an appropriate number of patients with a sufficiently long follow-up period for providing a robust comparison of the efficacy of the two ablation methods. One such study is FreezeAF, which demonstrated non-inferiority of CB to RF at both 6 and 12 months post-procedure [16]. The primary endpoint of freedom from AF, with no persistent complications, was achieved by 73% and 75% of the CB and RF groups (per-protocol [PP]), respectively, at 12 months (*p* < 0.001 for non-inferiority). Non-inferiority was also observed in the FIRE AND ICE trial, where 1-year Kaplan–Meier estimates for clinical failure after 12 months were reported to be 34.6% and 35.9% for CB and RF, respectively (*p* < 0.0001 for non-inferiority) [8, 17]. In the Cryo Versus RF trial, procedural success was reported to be higher for CB ablation compared to RF, with single-procedure success rates at 12 months being 76% and 47%, respectively (*p* < 0.001) [18].

These data demonstrate the potential of CB ablation for the treatment of paroxysmal AF; however, long-term follow-up evidence is lacking. Here, we present data collected at 30 months post-procedure in the FreezeAF trial.

## Methods

FreezeAF is a prospective randomised controlled trial designed to assess whether pulmonary vein isolation (PVI) with the Arctic Front Cardiac CryoAblation Catheter System (Medtronic, Inc., Minneapolis, MN) is non-inferior to that achieved using standard RF ablation [19]. Random number generation software was utilised to assign patients to one or other of the PVI techniques. The first patient was included on October 20, 2008. Following the procedure, there was a blanking period of 3 months. Follow-up clinical visits were then conducted at 3, 6, and 12 months. After completion of the follow-up within the scope of the trial, continued clinical visits and Holter ECG recordings were performed in our outpatient department on a yearly basis. Patients who refused to come to a follow-up were contacted by telephone and ECG documentation was obtained by the primary care physician. For patients lost to follow up, information whether the patient was still alive was obtained from the primary care physician. For this analysis a 30 month follow-up was performed.

All patients that were included in the study provided written informed consent. The trial was approved by the ethics committee at the University of Freiburg (September 15th, 2008), and was conducted in accordance with the Declaration of Helsinki and its amendments.

### Patients

Patients that had experienced at least two episodes of paroxysmal AF (of which at least one was documented) within the 3 months prior to enrolment were included. Further inclusion criteria included an age of between 18 and 75 years, and documented inefficacy of at least one AAD. Patients were excluded if they presented with a left atrium of >55 mm, displayed evidence of left atrial thrombus, had previously undergone left atrial surgery or ablation, had an ejection fraction of <40%, had heart failure of New York Heart Association (NYHA) class III or IV, had a mitral prosthesis, had suffered a myocardial infarction (MI) within the previous 3 months, had undergone percutaneous coronary intervention (PCI) or cardiac surgery within the previous 3 months, had suffered a stroke or transient ischaemic attack (TIA) within the previous 6 months, were pregnant, or had a life expectancy of less than 1 year. A full list of inclusion and exclusion criteria has previously been published [19].

### Procedures

Transoesophageal echo was routinely performed in patients undergoing left-atrial ablation and independent of any potential previous anticoagulation. It was also routinely performed in patients undergoing right-atrial ablation but without a 4-week course of anticoagulation (VKA, NOAC).

A detailed description of the ablation procedures that were performed has been published previously [16, 19]. Briefly, RF ablation was carried out using an irrigated tip catheter in conjunction with a 3D navigation system (Ensite NavX/Velocity, St Jude Medical, St Paul, MN; CARTO-3, Biosense Webster Inc., Diamond Bar, CA). CB ablation was primarily performed using the Arctic Front Cardiac CryoAblation Catheter System and the FlexCath Steerable Sheath (Medtronic, Minneapolis, MN). A small number of procedures were performed using the second generation Arctic Front Advance. A 28 mm balloon was used preferentially, although a change to a 23 mm balloon was permitted if deemed necessary. The investigator identified the presence of a common ostium of the left sided based on angiography.

All patients received anticoagulation (VKA or NOAC) throughout the 4 weeks prior to the index procedure and the subsequent 6 months. VKAs were bridged with subcutaneous heparin was used if patients were receiving phenprocoumon and had an international normalised ratio (INR) of <2; otherwise (INR >2), VKAs were not interrupted. DOACs were not interrupted in any case and also given in the morning prior to ablation. AADs were discontinued 4 to 5 half lives prior to the ablation procedure, and beta blockers were the only drugs administered following it.

Patients were followed in 6 months intervals on an ambulatory basis. The visit included a 7-day Holter electrocardiogram. In case the patient was not able to attend the visit, the information were requested from the treating general practitioner/office based cardiologist. Further ablation procedures could be carried out after 6 months had elapsed if deemed necessary. In these cases, the same energy source as that used for the index procedure was employed.

### Outcomes

Procedural success was defined as freedom from AF, in combination with an absence of persistent complications (after the 3-month blanking period). Persistent complications were defined as those classed as major or symptomatic, and that remained present 6 months after the procedure. Outcomes were assessed with regard to the success of a single procedure, and as success after one or more procedures. Procedural characteristics and safety outcomes were analysed at 6 and 12 months, and have been published elsewhere [16].

### Statistics

The evaluation criteria single as well as multiple procedure success were evaluated for the intention-to-treat (ITT) population.

The test for non-inferiority was formulated in terms of the rate of procedural success at the follow-up point for the patients that had undergone the CB procedure (P_CB_) and those that had undergone the RF procedure (P_RF_). The null hypothesis (H_0_) was thereby defined as: P_CB_ – P_RF_ ≤ −δ, where δ = 0.15 [19]. The non-inferiority test for rates according to Farrington and Manning was performed at a one-sided significance level of α = 2.5% [20].

Data are presented as means with standard deviations (SD), medians with first and third quartiles, or absolute values with percentages. Multiple logistic regression analysis was used to assess the influence of both clinical as well as procedure-specific factors on single-procedure success. To this end logistic regression models were fitted for the overall population, the RF group (including RF specific covariates) and the CB group (including CB specific covariates). Due to the multitude of factors of interest and the resulting instability of standard logistic regression, we used Firth’s penalized logistic regression to obtain stable results [21]. Statistical analysis was performed using R v3.13 and the ‘mice’ package (Multivariate Imputation by Chained Equations) v2.22 [22]. All model variables were included in the multiple imputation procedure (full predictor matrix) to minimise bias from the imputation method and 10 datasets were imputed.

## Results

**Fig. 1:**
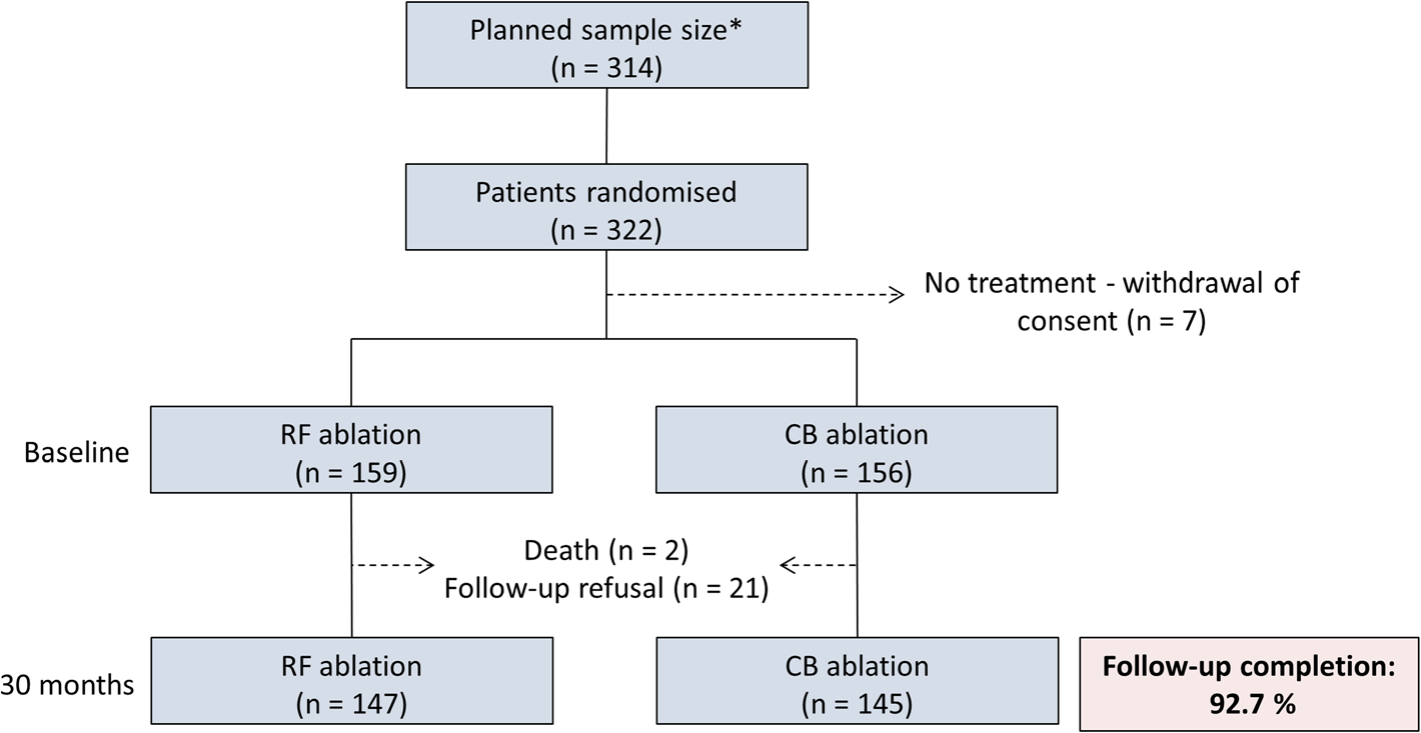
Patient flow, including planned sample size recalculation. *The initial sample size calculation resulted in 244 patients, which was readjusted in March 2011 after a pre-specified blinded sample size recalculation [16, 33]. Patients withdrawing consent prior to treatment were unaware of the assignment and were excluded from the population. Patients that refused a follow-up were contacted by phone to verify that they were alive, though no heart rhythm was obtained. RF, radiofrequency; CB, cryoballoon

Of the 315 patients that were randomised and received catheter ablation, 292 (92.7%) completed the 30-month follow-up (Fig. 1). The median age of the patients was 61.0 years, and 60.3% were male (Table 1). The baseline characteristics of the RF group (*N* = 147) and the CB group (*N* = 145) did not differ significantly, although a common ostium was slightly more prevalent in the RF group compared to CB (23.8% vs. 13.8%; *p* = 0.04). A total of 73.3% of patients had been treated with phenprocoumon, while 26.0% were taking a direct acting oral anticoagulant (DOAC) and 11.9% were additionally taking a platelet inhibitor. Again, there were no significant differences between the RF and CB groups. Further details on the baseline characteristics, medications, and risk indices (CHA_2_DS_2_-VASc, HAS-BLED) can be found in our previous report, along with data concerning periprocedural complications [16]. Briefly, the occurrence of vascular events (*p* = 0.372) and pericardial effusion (*p* = 0.683) did not vary between the RF and CB groups, while phrenic nerve palsy was observed exclusively in the CB group (3 major events, 6 minor events).

**Table 1:** Patient characteristics

	Total (*N* = 292)	RF (*N* = 147)	CB (*N* = 145)	*p*-value RF vs. CB
Male gender	176 (60.3)	83 (56.5)	93 (64.1)	0.22
Age (years)^†^	61.0 (54.8, 67.0)	60.0 (55.0, 67.5)	62.0 (54.0, 66.0)	0.96^‡^
Comorbidities				
CAD	37 (12.7)	18 (12.2)	19 (13.1)	0.83^†^
Hypertension	187 (64.0)	95 (64.6)	92 (63.4)	0.93
Diabetes	28 (9.6)	16 (10.9)	12 (8.3)	0.58
Vascular disease	15 (5.1)	11 (7.5)	4 (2.8)	0.12
Common ostium	55 (18.8)	35 (23.8)	20 (13.8)	0.04
Prior medication				
Phenprocoumon	214 (73.3)	108 (73.5)	106 (73.1)	1.00
DOAC	76 (26.0)	37 (25.2)	39 (26.9)	0.84
Platelet inhibitor^a^	34 (11.9)	21 (14.7)	13 (9.2)	0.20
Bridging^b^	40 (14.1)	24 (16.9)	16 (11.3)	0.23

^†^Data given as median (p25, p75)

^‡^P-value calculated using Mann-Whitney U-test. All other *p*-values calculated using chi-square test

^a^N: total, 286; RF, 143; CB, 142

^b^N: total, 284; RF, 142; CB, 142. CAD, coronary artery disease; DOAC, direct oral anticoagulant

**Fig. 2:**
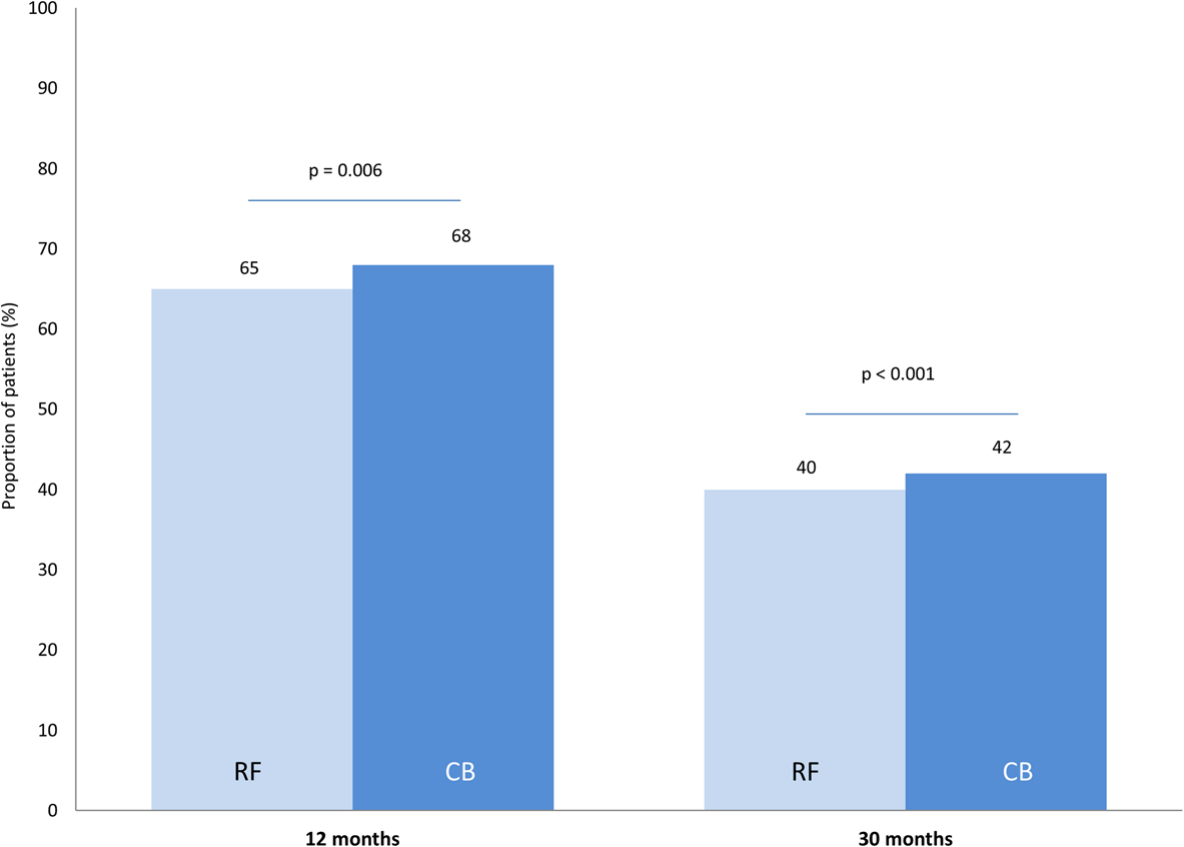
Freedom from AF, without persistent complications (ITT analysis, single procedure success). RF: *N* = 147; CB: *N* = 145. *P*-values are for non-inferiority

**Fig. 3:**
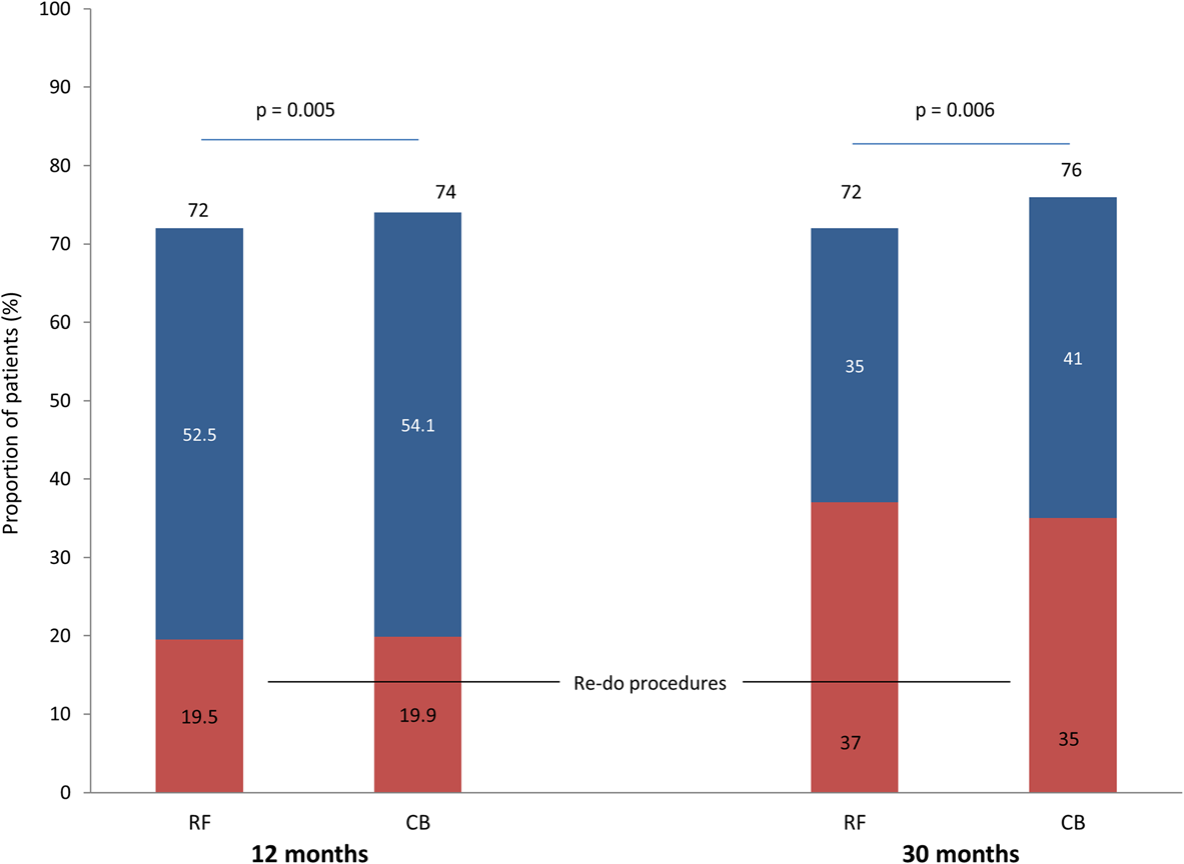
Freedom from AF, without persistent complications (ITT analysis, multiple procedure success). Thirty-month analysis includes 6 CB patients who underwent RF as a re-do procedure. No RF patients had a CB re-do procedure. *P*-values are for non-inferiority

Procedural success, defined as freedom from AF with an absence of persistent complications, at 30 months after the index procedure was achieved by 40% of patients in the RF group and 42% of the CB group (*p* < 0.001 for non-inferiority; Fig. 2). When taking multiple procedures into account, procedural success was 72% and 76% for the RF and CB groups, respectively (*p* = 0.006 for non-inferiority; Fig. 3). The proportions of patients that had undergone re-do procedures in order to achieve procedural success increased from the 12-month to the 30-month follow-up point, reaching 37% and 35% for the RF and CB groups, respectively. It should be noted that between 12 and 30 months, six patients in the CB group underwent a re-do procedure using RF (based on a random decision at the time of recurrence), while none of the RF group underwent a CB procedure. After the first six patients crossed over a decision was made to treat subsequent patients according to their group assignment for recurrences.

The only variable that was found to be predictive of single procedure success was being male (Table 2). However, this was only true for the overall population (OR: 3.42; 95% CI: 1.09–10.71; *p* = 0.04) and the RF group (OR: 9.88; 95% CI: 1.36–70.08; *p* = 0.02). The type of procedure was not predictive of success (OR: 1.02; 95% CI: 0.45–2.32; *p* = 0.96) which is in line with the significant result of the non-inferiority test. The use of the second generation balloon ((Arctic Front Advance Cardiac CryoAblation Catheter System (Medtronic Inc., Minneapolis, MN)) appeared to be predictive of improved efficacy; however, due to the later availability and the resulting small number of procedures (*n* = 22) the confidence interval was extremely large (OR: 9.77; 95% CI: 0.30–320.56; *p* = 0.20) (for details see Table 3). Of the 22 procedures conducted with the new balloon, 17 were successes while only 42 of the 120 procedures with the old balloon were successful after the first procedure. With the use of the second generation balloon the mean procedure time was reduced from 170.5 min to 103.2 min, which corresponds to a mean difference of 67.3 min (95% CI: 49.7–84.9 min; *p* < 0.0001). There also was a reduction of the fluoroscopy duration (27.2 vs. 18.6 min; mean difference 8.6 min; 95% CI: 3.8–13.2 min; *p* = 0.0004).

**Table 2:** Adjusted odds ratios^a^ for achievement of single procedure success

	Total (*N* = 292)		RF (*N* = 147)		CB (*N* = 145)	
	OR (95% CI)	*p*-value	OR (95% CI)	*p*-value	OR (95% CI)	*p*-value
Group: CB	1.02(0.45–2.32)	0.96	N/A	N/A	N/A	N/A
Age	1.02 (0.96–1.08)	0.50	1.05 (0.95–1.17)	0.31	1.00 (0.95–1.07)	0.90
Gender: male	3.42 (1.09–10.71)	0.04	9.77 (1.36–70.08)	0.02	2.12 (0.70–6.42)	0.19
CAD	1.32 (0.25–6.96)	0.74	1.93 (0.12–30.67)	0.64	1.18 (0.17–8.06)	0.87
Hypertension	0.53 (0.19–1.51)	0.24	0.11 (0.01–1.33)	0.08	0.85 (0.30–2.40)	0.76
Diabetes	0.72 (0.22–2.43)	0.60	0.63 (0.08–4.86)	0.66	0.94 (0.20–4.50)	0.94
Vascular disease	0.57 (0.07–4.37)	0.59	0.47 (0.02–14.38)	0.66	0.30 (0.01–6.06)	0.43
Common ostium	1.36 (0.42–4.40)	0.60	2.26 (0.31–16.57)	0.42	1.11 (0.25–4.98)	0.89
Bridging	0.92 (0.32–2.65)	0.88	0.74 (0.15–3.72)	0.72	1.57 (0.31–8.08)	0.59
Platelet inhibitor	0.81 (0.20–3.31)	0.77	0.52 (0.08–3.57)	0.51	1.08 (0.14–8.01)	0.94
DOAC vs. Phenprocoumon	3.91 (0.30–50.12)	0.29	3.14 (0.24–40.93)	0.28	1.21 (0.31–4.66)	0.78
Follow-up time	0.92 (0.75–1.13)	0.44	1.01 (0.82–1.24)	0.94	0.90 (0.67–1.20)	0.47
NavX navigation system	N/A	N/A	0.37 (0.06–2.12)	0.27	N/A	N/A
Balloon size: 23 mm	N/A	N/A	N/A	N/A	1.89 (0.44–8.07)	0.39
Balloon size: both	N/A	N/A	N/A	N/A	0.65 (0.03–12.44)	0.78
Second generation balloon	N/A	N/A	N/A	N/A	9.77 (0.30–320.56)	0.20
Achieve catheter	N/A	N/A	N/A	N/A	1.24 (0.38–4.10)	0.72
Multiple balloons	N/A	N/A	N/A	N/A	1.26 (0.10–16.20)	0.86

^a^Adjusted for all applicable variables. N/A: not applicable; CAD, coronary artery disease. Procedural success defined as freedom from AF with an absence of persistent complications, at 30 months

**Table 3:** Subgroup analyses CB-1 versus 2

	CB – 1 (*N* = 120)	CB – 2 (*N* = 22)	*p*-value
Isthmus ablation (RF)	15 (13%)	4 (18%)	0.61
Common ostium	15 (13%)	5 (23%)	0.35
Total procedure time (min)	170.5	103.2	<0.001
X-ray duration (min)	27.2	18.6	0.0004
X-ray dose (cGy*cm^2^)	77.4	55.0	0.10
Single procedure success	42 (35%)	17 (67%)	0.001

CB-1: first generation Arctic Front; CB-2: second generation Arctic Front Advance (both Medtronic, Minneapolis, MN)

## Discussion

The randomised FreezeAF trial was performed in order to evaluate the efficacy of CB ablation as an alternative to the established RF approach. After an extended follow-up of 30 months, CB ablation was non-inferior to RF in terms of freedom from AF, without persistent complications.

PV isolation using a CB has a number of potential advantages to that using RF ablation. Firstly, the overall procedure time is generally lower, owing to the one-step lesion-formation process. Indeed, in the FreezeAF trial, the median procedure time was 174 min for the RF group and 161 min for the CB (*p* = 0.006) [16]. This is in agreement with the FIRE AND ICE study, where procedure duration was 140.9 min for RF and 124.4 min for CB (*p* < 0.001) [8]. Although the duration of fluoroscopy was comparable between the two procedure types in FreezeAF, CB required a higher X-ray dose [16]. However, in the present analysis, a comparison of the first and second generation CBs showed not only a significant reduction in fluoroscopy time with the newer model, but also a slightly lower X-ray dose. While only a small number of procedures were performed using the second generation CB, other recent studies have also demonstrated significant advantages over the earlier version in terms of both procedure time and fluoroscopy time [23, 24]. Further studies involving the later generations of RF and CB devices are needed in order to determine if there remains significant differences in procedural duration and fluoroscopy between them.

There is a growing body of evidence from registries [6, 25] and clinical studies [11–15] demonstrating that CB ablation is similar in efficacy to RF; however, there are only limited data from randomised controlled trials. We previously demonstrated that procedural success with the CB method was non-inferior to that with the RF, at both 6 and 12 months post-ablation [16]. Furthermore, periprocedural complication rates were not significantly different between the two methods, with the exception of a higher incidence of phrenic nerve palsy in the CB patients [16]. Here, we have re-evaluated the original primary endpoint to include the patients with efficacy information available after 30 months. The finding of non-inferiority was maintained after this extended period of time. These data are in agreement with those obtained in the randomised FIRE AND ICE study, where Kaplan–Meier estimates of procedural failure were calculated to be 35.9% and 34.6% at 1 year, respectively (*p* < 0.001 for non-inferiority) [8, 17]. While the maximum follow-up time was 33 months in this case, patient numbers declined greatly after the first year, with the mean follow-up being 1.5 years. In FreezeAF, 92.7% of the patients that underwent the index procedure completed the 30-months follow-up period, providing a robust estimate of long-term procedural success.

Multivariable analysis demonstrated that being male was predictive of single procedure success at 30 months for patients undergoing the RF procedure, although the confidence interval was large. In the Cryo Versus RF trial, female gender was identified as being predictive of recurrent atrial arrhythmia after adjustment for the ablation method used (HR: 2.22; 95% CI: 1.22–4.01; *p* = 0.009) [18]. As in our study, Neumann et al. found no association between gender and procedural success when using the CB technique [26]. Out of all the variables assessed in our analysis, none were found to have substantial predictive value for single procedure success. This included procedural characteristics such as the use of the NavX 3D navigation system as opposed to the CARTO (RF procedure), and use of the Achieve mapping catheter as opposed to the Lasso (CB procedure). For the CB procedure, use of the second generation cryoballoon appeared to increase the likelihood of procedural success; however, the confidence interval was extremely large. It is possible that with a higher number of patients, this difference could be confirmed to be statistically significant. As the present study was initiated in 2008, the majority of subjects underwent ablation using the first generation balloon, preventing us from accurately comparing the different models. Some recent studies have found that procedure time appears to be shorter with the Advance model, while long-term freedom from AF is superior [23, 24]. One study found that the rate of phrenic nerve palsy was higher with the second generation CB, [27] while other studies have reported no difference [23, 24]. Improvements in RF systems have also been introduced in recent years, the main development being contact force sensing [28, 29]. A limitation to the present study is that at the time of its initiation, contact force systems were not yet available. The analysis is therefore primarily a comparison of the first generation RF and CB devices, albeit with a small number of second generation CB procedures included. The FIRE AND ICE study had a similar limitation, with first and second generations of both types of device used [8]. In fact, the majority of patients in the RF group received treatment with the first generation device, while for the CB procedure, there were significantly more second generation devices used. Notably, there are early indications that the non-inferiority of CB compared to RF could be maintained for the later generations of catheter ablation systems [13, 15, 30]. However, at present, there are no long-term data available concerning the efficacy of the newer systems. Comparisons of the early models therefore provide a valuable indication of the long-term success of the two ablation techniques.

One potential source of bias in the comparison of the long-term outcomes of RF and CB ablation is the higher prevalence of a common ostium that was observed in the RF group. As the CB approach is potentially more difficult in the presence of this defect, procedural success rates may have been affected by this. However, the presence of a common ostium was not found to be associated with procedural success in the multivariate analysis, in either group, which is in agreement with other studies [31, 32].

## Conclusions

The therapeutic non-inferiority of CB ablation for the treatment of paroxysmal AF has again been demonstrated. In comparison with the traditionally-used RF energy source, the cryo technique was found to be non-inferior, which is in agreement with other studies. Furthermore, the second generation balloon appeared to be associated with more procedures being successful after the first attempt than the previous model. We have shown here that this non-inferiority continues long-term, with the achievement of freedom from AF being high 30 months after the index procedure. This extended follow-up of the FreezeAF trial provides evidence that the CB should be considered as a viable alternative to RF ablation for patients with paroxysmal AF.

## Abbreviations

AAD: Antiarrhythmic drugs
AF: Atrial fibrillation
CB: Cryoballoon
DOAC: Direct acting oral anticoagulant
ITT: Intention-to-treat
MI: Myocardial Infarction
NYHA: New York Heart Association
PCI: Percutaneous intervention
PP: Per protocol
PV: Pulmonary veins
PVI: Pulmonary vein isolation
RF: Radiofrequency
TIA: Transient ischemic attack
